# The Last Plague or Before the Graying

**DOI:** 10.3201/eid2610.202788

**Published:** 2020-10

**Authors:** Ronald O. Valdiserri

**Affiliations:** Emory University Rollins School of Public Health, Atlanta, Georgia, GA

**Keywords:** Antibodies, blood tests, coronavirus disease, COVID-19, HIV/AIDS, plague, SARS-CoV-2, viruses, severe acute respiratory syndrome coronavirus 2, zoonoses

When the last plague struck adulthood was new.
Youth *finis—*but not so far behind that one couldn’t feel its humming.
AIDS stained everything with sorrow, yes, but it also fired action.
Those years the only verbs I breathed were *demand, confront, claim*!
Christened by a blood test that found no antibodies,
We lucky ones were labeled “negative.”
An ironic nomenclature: deemed HIV-free despite being seized by the disease.
Scorched by anger ignited through society’s indifference.
Blazing to fight against the epidemic, each in his own way.
Quietly, as “buddies,” tendering service in shaded sick rooms,
Or loudly, through defiant pageants of outrage hurled in public.
Never doubting our capacity to beat-back the epidemic.
But that was before the graying, when possibilities measured time.
Now, on maturity’s leeward slope, comes a new plague, a different virus.
SARS-CoV-2, the unrelenting agent of COVID-19:
Inescapable television image, societal stopwatch, economic paralytic.
Unlike HIV in biography and in its command of instant global attention.
Different, too, my reaction: scrappy resolve replaced now by enervation.
And I wonder, do mounting years explain this change or could it be the times?
Whence the source: the inevitable stiffening of age or pessimism’s bloodless clinch?
Dr. Valdiserri is a professor in the Department of Epidemiology, Rollins School of Public Health, Emory University, Atlanta, GA. He previously held senior leadership positions at the Centers for Disease Control and Prevention, the US Department of Veterans Affairs, and the Office of the Assistant Secretary for Health, Department of Health and Human Services. He has written extensively on the public health aspects of HIV/AIDS, sexually transmitted infections, and viral hepatitis.
Address for correspondence: Ronald O. Valdiserri, Emory University, 201 Dowman Dr, Atlanta, GA 30322, USA; email: rvaldis@emory.edu

